# Prognostic Nutritional Index as a Predictor of 90-Day Mortality in Surgical Sepsis Patients with Acute Kidney Injury—A Retrospective Cohort Study Based on the MIMIC-IV Database

**DOI:** 10.3390/jcm15145706

**Published:** 2026-07-21

**Authors:** Jia Wan, Chaoqun Zhang, Kuncan Lin, Tiehua Li, Yong Huang, Xiaolong Ye

**Affiliations:** 1 Department of Internal Medicine ICU, The Third Affiliated Hospital of Sun Yat-sen University, Guangzhou 510630, China; wanj23@mail.sysu.edu.cn (J.W.); litiehua@mail.sysu.edu.cn (T.L.); 2 Department of General ICU, The Third Affiliated Hospital of Sun Yat-sen University Zhaoqing Hospital, Zhaoqing 526070, China; lz070129zcq@outlook.com (C.Z.); lkc5139@outlook.com (K.L.); 3 Department of Gastrointestinal Surgery, The Third Affiliated Hospital of Sun Yat-sen University, Guangzhou 510630, China; 4 Department of Gastrointestinal Surgery, The Third Affiliated Hospital of Sun Yat-sen University Zhaoqing Hospital, Zhaoqing 526070, China

**Keywords:** prognostic nutritional index, sepsis, acute kidney injury, surgical intensive care unit, mortality, MIMIC-IV

## Abstract

**Background:** Surgical sepsis complicated by acute kidney injury (AKI) is associated with high mortality, and early risk stratification remains challenging. The prognostic nutritional index (PNI), derived from serum albumin and lymphocyte count, reflects nutritional and immune status, but its prognostic value in surgical sepsis with AKI has not been well defined. **Methods:** In this retrospective cohort study based on the Medical Information Mart for Intensive Care IV (MIMIC-IV) (version 3.1) database, we included adult patients admitted to a surgical or surgery-related intensive care unit (ICU) between 2008 and 2022 who met the Sepsis-3 criteria and developed AKI according to KDIGO. The primary endpoint was 90-day all-cause mortality. We used multivariable Cox proportional hazards models, restricted cubic splines, Kaplan–Meier analysis, and predefined subgroup analyses to examine the association between PNI at ICU admission and 90-day mortality. **Results:** A total of 1483 patients were included, with a 90-day mortality rate of 30.2%. Non-survivors had a lower median PNI than survivors (35 vs. 36, *p* < 0.001). After sequential adjustment for demographics, comorbidities, illness severity, and major interventions, each 1-point increase in PNI was associated with a 1.5% reduction in 90-day mortality (hazard ratio 0.985, 95% confidence interval 0.974–0.997, *p* = 0.012). Restricted cubic spline analysis showed an approximately linear inverse relationship between PNI and mortality risk (*p* for overall association = 0.046; *p* for nonlinearity = 0.534). Using an optimal cut-off of 29.51, patients with low PNI had significantly lower 90-day survival than those with high PNI (log-rank *p* < 0.0001), and subgroup analyses demonstrated generally consistent protective associations across age, sex, illness severity, and key treatments. In propensity score-matched analysis, the association was attenuated and no longer significant (HR = 1.174, 95% CI: 0.901–1.529, *p* = 0.234), although the direction of effect remained consistent. **Conclusions:** Lower PNI at ICU admission was associated with higher 90-day mortality in multivariable-adjusted models and may serve as a simple, routinely available adjunctive marker for early risk stratification in surgical sepsis patients with AKI. However, given the non-significant finding in propensity score-matched analysis, its independent prognostic value remains uncertain, and further prospective studies are needed to validate its clinical utility.

## 1. Introduction

Sepsis is one of the most challenging syndromes in critical care medicine. It is essentially an uncontrolled inflammatory and immune response to infection, leading to progressive organ dysfunction and high mortality [1,2,3]. Acute kidney injury (AKI) is among the most common complications of sepsis, and once it develops, both in-hospital and long-term mortality increase markedly [4,5]. In the surgical intensive care unit (SICU), patients are subjected to surgical trauma, infectious stress, and a highly catabolic state. The kidneys are particularly vulnerable to hemodynamic instability, inflammatory “storms,” and nephrotoxic agents [6,7]. Early identification of high-risk patients with poor prognosis is therefore crucial to the management of surgical sepsis complicated by AKI.

Malnutrition and immune dysregulation are highly prevalent in critically ill patients and are closely related to the onset and progression of sepsis-associated AKI [8,9]. Current guidelines advocate systematic nutritional risk screening and individualized nutritional interventions in critically ill patients [10,11]. However, traditional nutritional assessment tools (e.g., NRS 2002 and Subjective Global Assessment) are cumbersome and exhibit poor reproducibility in the rapidly changing setting of critical illness. On the other hand, although numerous markers of immune status have been proposed, their testing is often costly and difficult to implement at scale in routine clinical practice. Immunocompromised status has been identified as an independent risk factor for mortality in sepsis patients, with a recent prospective cohort study reporting a nearly three-fold increased risk of death [12].

The prognostic nutritional index (PNI), first proposed by Onodera et al. [13], combines serum albumin levels and peripheral blood lymphocyte counts. It reflects not only synthetic capacity and internal homeostasis but also, to some extent, the host immune status [14,15]. Recent studies have shown that PNI is closely associated with mortality in general sepsis or mixed ICU populations [16,17,18,19].

However, patients with surgical sepsis and AKI are highly heterogeneous. In addition to surgical factors such as perioperative fluid management, type of operation, and blood loss, they are also affected by the systemic inflammatory response and immune imbalance associated with sepsis. In this specific population, the prognostic value of PNI has not been systematically examined using large-scale, real-world data. Therefore, we used the MIMIC-IV database [20,21] to identify adult patients with surgical sepsis and AKI and systematically analyzed the relationship between PNI at ICU admission and 90-day all-cause mortality, with particular emphasis on its dose–response pattern and stability across subgroups. Our aim was to explore the association between PNI at ICU admission and 90-day mortality in this high-risk population, and to provide preliminary evidence for its potential role in early risk stratification.

## 2. Materials and Methods

### 2.1. Data Source and Ethical Approval

Data for this study were obtained from the MIMIC-IV (Medical Information Mart for Intensive Care IV, version 3.1) database, which contains information on adult patients admitted to the ICUs of Beth Israel Deaconess Medical Center (Boston, MA, USA) between 2008 and 2022 [20,21]. All patient data in MIMIC-IV are de-identified. One of the authors (Xiaolong Ye) completed the National Institutes of Health’s web-based course “Protecting Human Research Participants” (Record ID: 71643049) and was approved to access the database to extract data. All data in this database were deidentified, leading the Ethics Committee of Beth Israel Deaconess Medical Center to grant a waiver of informed consent. This study adheres to the Strengthening the Reporting of Observational Studies in Epidemiology (STROBE) statement [22] and the principles of the Declaration of Helsinki.

### 2.2. Study Population

Patients were included if they met all of the following criteria:

- First ICU admission was to a surgical or surgery-related ICU, including surgical ICU, cardiothoracic ICU, neurosurgical ICU or trauma surgical ICU;
- The patient’s condition met the Sepsis-3 definition: suspected or confirmed infection plus an increase in SOFA score of ≥2 points from baseline;
- The patient developed AKI during ICU stay, defined according to Kidney Disease: Improving Global Outcomes (KDIGO) criteria, based on serum creatinine and/or urine output [23].

AKI was identified using the MIMIC-IV KDIGO stages table, which applies KDIGO criteria throughout the ICU stay. Patients with a maximum KDIGO stage ≥ 1 during the ICU admission were considered to have AKI, regardless of whether AKI was present at admission or developed subsequently.

The KDIGO stages table incorporates both serum creatinine criteria (≥0.3 mg/dL increase within 48 h or ≥1.5-fold increase over 7 days) and urine output criteria (<0.5 mL/kg/h for ≥6 h), using all available creatinine and urine output measurements to assign KDIGO stages according to the standard MIMIC-IV KDIGO implementation.

Patients were excluded if they:

- Were younger than 18 years;
- Had an ICU length of stay < 24 h;
- Lacked serum albumin or lymphocyte count measurements within the first 24 h after ICU admission, precluding calculation of PNI;
- Had multiple ICU admissions during the same hospitalization, in which case only the first ICU stay was retained.

### 2.3. Variable Extraction and Calculation of PNI

Structured query language (SQL) was used to extract the following data from MIMIC-IV:

- Demographics: age, sex, body weight, etc.;
- Vital signs within 24 h after ICU admission: systolic blood pressure, mean arterial pressure, heart rate, respiratory rate, etc.;
- Laboratory tests: serum albumin, peripheral blood lymphocyte count, white blood cell count, lactate, serum creatinine, electrolytes, total bilirubin, blood urea nitrogen (BUN), red cell distribution width (RDW), and others;
- Severity scores: SOFA score and related variables;
- Therapeutic interventions: use of vasopressors, continuous renal replacement therapy (CRRT), mechanical ventilation, etc.;
- Outcomes: 90-day all-cause mortality, determined by combining in-hospital electronic records and follow-up information.

PNI was calculated as 10 × serum albumin (g/dL) + 5 × total lymphocyte count (10^9^/L) [13]. Serum albumin and lymphocyte count values were taken from the first available tests performed within 24 h after ICU admission. SOFA scores were calculated using the worst value of each component during the first 24 h after ICU admission. If a component was not measured within this window, it was scored as 0.

### 2.4. Endpoint

The primary endpoint was 90-day all-cause mortality. Time zero was defined as the time of first ICU admission. Patients were followed until death or 90 days after ICU admission, whichever came first.

### 2.5. Statistical Analysis

Continuous variables were expressed as mean ± standard deviation or median (inter-quartile range, IQR) as appropriate and compared between groups using the *t*-test or Mann–Whitney U test. Categorical variables were expressed as counts and percentages and compared using the χ^2^ test or Fisher’s exact test.

To examine the association between the prognostic nutritional index (PNI) and 90-day mortality, multivariable Cox proportional hazards models were constructed. PNI was treated as a continuous variable, and the hazard ratio (HR) with 95% confidence interval (CI) was estimated. Models were adjusted stepwise for demographic variables, comorbidities, and key clinical factors. The dose–response relationship was explored using restricted cubic splines (RCS) with 4 knots to assess overall and nonlinear associations between PNI and the log HR of mortality [24].

Propensity score matching (PSM) was performed to balance baseline characteristics between low-PNI (PNI < 29.51) and high-PNI (PNI ≥ 29.51) groups using 1:1 nearest-neighbor matching with a caliper of 0.2 standard deviations of the logit of the propensity score. The propensity score model included age, SOFA score, lactate, creatinine, vasopressor use, and continuous renal replacement therapy (CRRT). Covariate balance was assessed using standardized mean differences (SMDs), with SMD < 0.1 indicating good balance. In the matched cohort, an unadjusted Cox model was first fitted for 90-day mortality, followed by an adjusted model that included any covariates with post-matching SMD ≥ 0.1 (notably lactate) to account for residual confounding.

Diagnostic tests for the Cox models included evaluation of the proportional hazards assumption using Schoenfeld residuals (a *p* value > 0.05 indicated no violation) and assessment of multicollinearity using the variance inflation factor (VIF), with VIF < 5 indicating no significant collinearity.

The optimal cut-off point for PNI was identified using the surv_cutpoint function in the R package “survminer” [25], which identifies the cut-off that maximizes the log-rank test statistic (i.e., minimizes the *p*-value) for the difference in survival between the two groups, with the constraint that each group contains at least 10% of the total sample. This cut-off should be considered exploratory and hypothesis-generating and requires external validation in independent cohorts before it can be applied as a clinical threshold. Patients were then classified into low- and high-PNI groups according to this cut-off. Kaplan–Meier curves were plotted, and the log-rank test was used to compare 90-day survival between the two groups.

Prespecified subgroup analyses were performed stratified by age, sex, race, SOFA score, mechanical ventilation, CRRT, vasoactive drugs, and the presence of major comorbidities (congestive heart failure, cerebrovascular disease, chronic pulmonary disease, diabetes, malignant cancer, severe liver disease). The consistency of the association between PNI and 90-day mortality across subgroups was assessed, and interaction terms were tested to explore potential effect modification.

For missing data, covariates with more than 20% missing values were excluded. Covariates with less than 20% missing data were handled using multiple imputation implemented in R software (version 4.2.3; The R Foundation, http://www.R-project.org).

All statistical analyses were performed using the same software. A two-tailed *p*-value of less than 0.05 was considered statistically significant. Reporting followed the Strengthening the Reporting of Observational Studies in Epidemiology (STROBE) statement [22].

## 3. Results

### 3.1. Patient Selection and Baseline Characteristics

**Figure 1:**
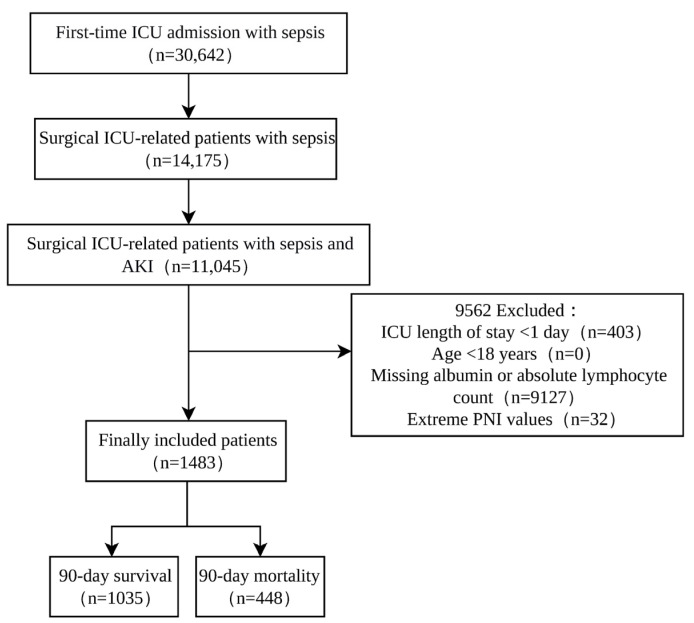
Flowchart of patient selection. Surgical or surgery-related ICUs include surgical ICU, cardiothoracic ICU, neurosurgical ICU, and trauma surgical ICU.

According to the inclusion and exclusion criteria, 1483 adult patients with surgical sepsis and AKI were identified from the MIMIC-IV database and included in the analysis. During 90-day follow-up, 448 patients died, corresponding to a cumulative 90-day mortality of 30.2% (Figure 1).

Baseline characteristics are shown (Table 1). Compared with survivors, non-survivors were older (70 vs. 64 years, *p* < 0.001) and had higher lactate (2.50 vs. 1.70 mmol/L, *p* < 0.001), lower albumin (2.90 vs. 3.00 g/dL, *p* = 0.016), and lower absolute lymphocyte count (0.90 vs. 1.07 × 10^9^/L, *p* < 0.001). Non-survivors also had higher SOFA scores (*p* = 0.002), were more likely to receive CRRT (28% vs. 9.9%, *p* < 0.001) and vasoactive drugs (68% vs. 57%, *p* < 0.001), and had higher prevalence of cerebrovascular disease (26% vs. 13%, *p* < 0.001). The median PNI was significantly lower in non-survivors (35 vs. 36, *p* < 0.001). Complete baseline characteristics, including vital signs and other laboratory parameters, are provided (Supplementary Table S1).

### 3.2. Association Between PNI and 90-Day Mortality in Multivariable Analysis

Cox proportional hazards models were used to assess the association between PNI and 90-day mortality. Univariable Cox regression analysis was performed as a preliminary screening (Supplementary Table S2). Higher PNI was significantly associated with lower 90-day mortality (per 1-point increase: HR = 0.981, 95% CI: 0.970–0.992, *p* < 0.001). Other factors associated with mortality included age, lactate, CRRT, vasoactive drugs, and several laboratory parameters (all *p* < 0.05).

To examine the association between PNI and 90-day mortality, we constructed four sequential multivariable Cox proportional hazards models (Table 2). Model 1 was unadjusted. Model 2 was adjusted for age, sex, and race. Model 3 further adjusted for illness severity indices (SOFA score, lactate, white blood cell count) and major comorbidities (congestive heart failure, diabetes, cerebrovascular disease, chronic pulmonary disease). Model 4 additionally included therapeutic interventions (vasopressor use and continuous renal replacement therapy, CRRT).

In the unadjusted Model 1, each 1-point increase in PNI was associated with a 1.9% reduction in mortality (HR = 0.981, 95% CI: 0.970–0.992, *p* < 0.001). Across the four sequential multivariable models, the association remained significant, with HRs ranging from 0.978 to 0.985 (all *p* < 0.05; Table 2). When expressed per 5-point increase in PNI, the HR was 0.927 (95% CI: 0.877–0.985, *p* = 0.012). The proportional hazards assumption was satisfied for PNI (*p* = 0.090; Supplementary Table S3), and no multicollinearity was detected (Supplementary Table S4).

### 3.3. Dose–Response Relationship Between PNI and Mortality

**Figure 2:**
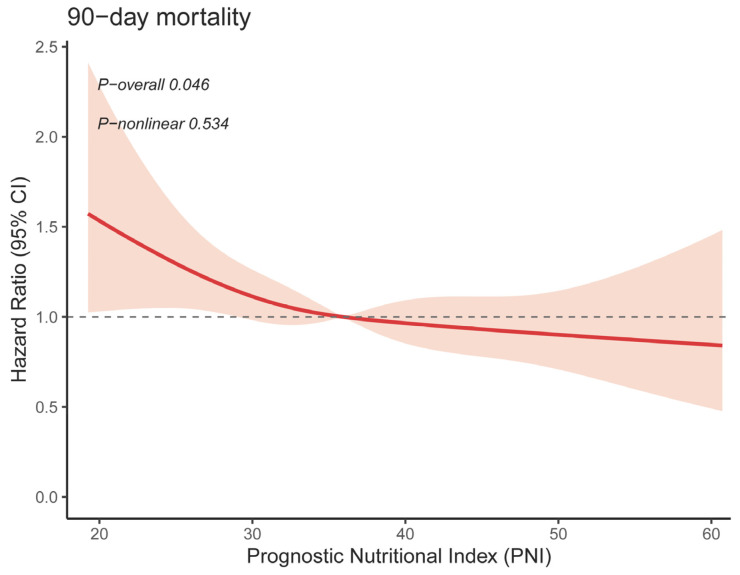
Restricted cubic spline curve for the association between PNI and 90-day mortality. The solid line represents the hazard ratio (HR), and the shaded area the 95% CI.

When PNI was modeled as a continuous variable using restricted cubic splines in the Cox model, a monotonically decreasing relationship between PNI and 90-day mortality was observed. As PNI increased, mortality risk decreased steadily, without evidence of a clear plateau or U-shaped curve within the commonly observed clinical range. The overall association was statistically significant (*p* for overall association = 0.046), whereas the test for nonlinearity was not (*p* for nonlinearity = 0.534), indicating that the relationship was predominantly approximately linear (Figure 2).

To further characterize the dose–response relationship, we categorized PNI into quartiles (Q1 to Q4, from lowest to highest). Using the highest quartile (Q4) as the reference, the lowest quartile (Q1) was associated with a significantly increased risk of 90-day mortality (HR = 1.424, 95% CI: 1.079–1.879, *p* = 0.013). A trend test across quartiles showed that each one-quartile increase in PNI was associated with a 10.3% reduction in mortality risk (HR = 0.897, 95% CI: 0.821–0.980, *p* = 0.016) (Table 3 and Supplementary Figure S1).

### 3.4. Survival Differences According to PNI Level

The optimal PNI cut-off value for predicting 90-day mortality was determined to be 29.51. Patients were accordingly divided into a low-PNI group (PNI < 29.51, n = 354) and a high-PNI group (PNI ≥ 29.51, n = 1129). Compared with the high-PNI group (Supplementary Table S5), patients in the low-PNI group had significantly higher heart rate, lactate, total bilirubin, RDW, PT, INR, and SOFA score, as well as lower albumin, lymphocyte count, hemoglobin, bicarbonate, and calcium (all *p* < 0.05). They were also more likely to receive CRRT or vasopressors (both *p* < 0.01), indicating a more severe clinical condition at ICU admission.

**Figure 3:**
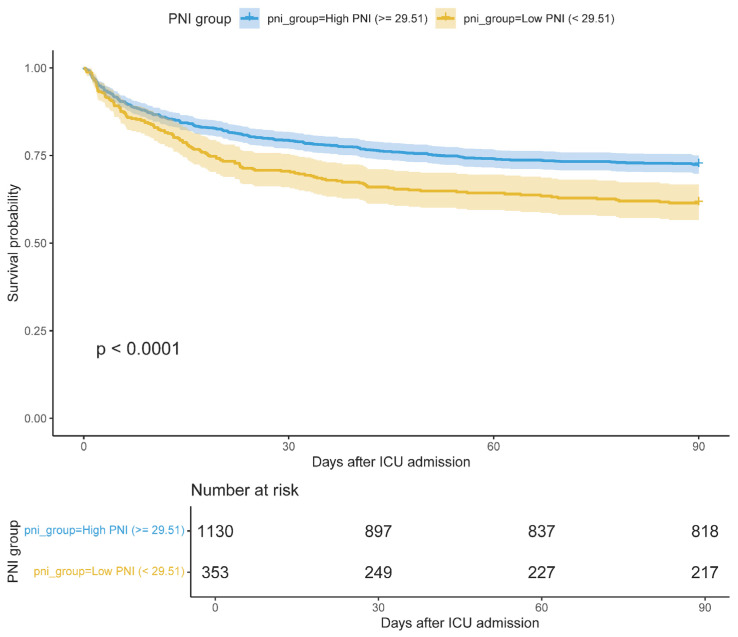
Kaplan–Meier survival curves for 90-day mortality in low- vs. high-PNI groups. Numbers at risk are shown below the x-axis.

Kaplan–Meier survival analysis showed that the survival curves of the two groups diverged early after ICU admission and remained separated throughout the 90-day follow-up (Figure 3). The low-PNI group had a significantly lower 90-day survival rate than the high-PNI group (log-rank *p* < 0.0001). After multivariable adjustment for age, sex, race, SOFA score, lactate, comorbidities, vasopressor use, and CRRT, low PNI remained significantly associated with an increased risk of 90-day mortality (HR = 1.351, 95% CI: 1.096–1.666, *p* = 0.005; Table 3). These findings suggest that the PNI cut-off of 29.51 may assist in risk stratification within this cohort, although this threshold requires external validation.

### 3.5. Subgroup Analyses

**Figure 4:**
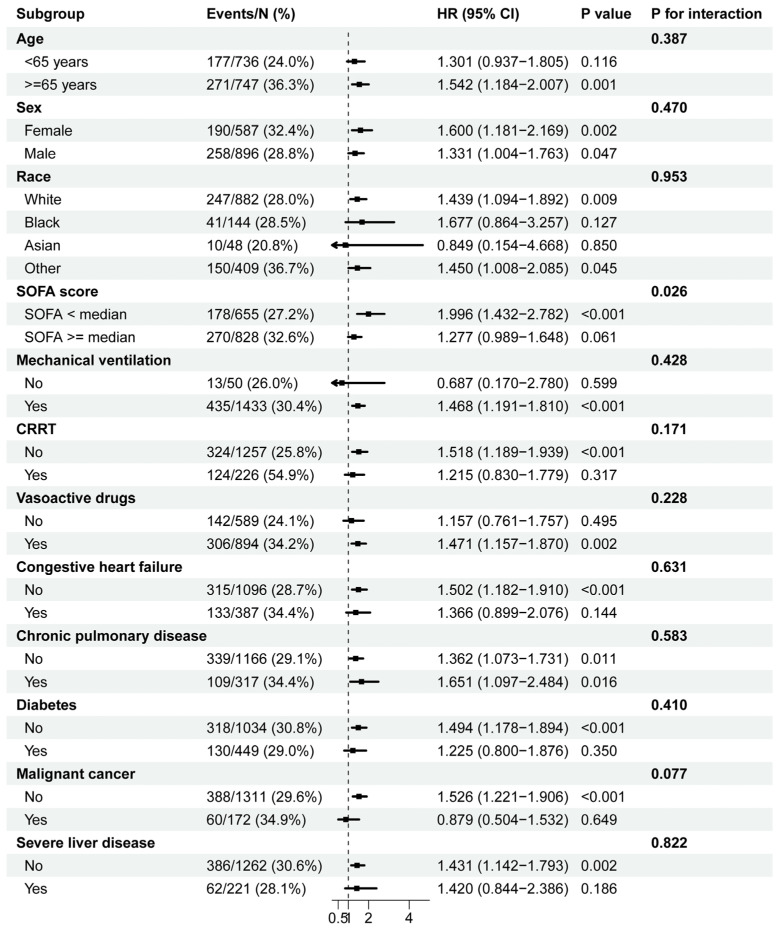
Subgroup analysis of the association between PNI and 90-day mortality. Subgroups: age, sex, race, SOFA score, mechanical ventilation, CRRT, vasoactive drugs, congestive heart failure, cerebrovascular disease, chronic pulmonary disease, diabetes, malignant cancer, severe liver disease. HRs and 95% CIs are shown.

Subgroup analysis showed that low PNI (PNI < 29.51) was consistently associated with higher 90-day mortality across all predefined subgroups, including age, sex, race, SOFA score, mechanical ventilation, CRRT, vasoactive drugs, and major comorbidities (Figure 4). The increased risk was more pronounced in patients with lower SOFA scores (SOFA < median: HR = 2.00, 95% CI 1.43–2.78) than in those with higher SOFA scores (HR = 1.28, 95% CI 0.99–1.65), and the interaction was statistically significant (*p* for interaction = 0.026). For most other subgroups, no significant interactions were observed, with *p* for interaction values of 0.387 (age), 0.470 (sex), 0.953 (race), 0.428 (mechanical ventilation), 0.171 (CRRT), 0.228 (vasoactive drugs), 0.631 (congestive heart failure), 0.583 (chronic pulmonary disease), 0.410 (diabetes), 0.077 (malignant cancer), and 0.822 (severe liver disease). These findings indicate that low PNI was consistently associated with higher mortality across most subgroups, with a stronger relative effect in patients who were less severely ill at ICU admission. This pattern across diverse patient groups highlights the potential value of PNI as an exploratory marker for early risk stratification, although its clinical utility requires further validation.

### 3.6. Propensity Score Matching Analysis

To further reduce confounding, we performed 1:1 propensity score matching based on age, SOFA score, lactate, creatinine, vasopressor use, and CRRT, generating 350 matched pairs of low-PNI (PNI < 29.51) and high-PNI (PNI ≥ 29.51) patients. Covariate balance before and after matching is shown in the Love plot (Supplementary Figure S2). After matching, most covariates were well balanced (standardized mean difference [SMD] < 0.1), with only lactate showing mild residual imbalance (SMD = 0.112).

In the matched cohort, low PNI was associated with higher 90-day mortality in the unadjusted Cox model (HR = 1.314, 95% CI: 1.021–1.692, *p* = 0.034). After additional adjustment for lactate, the effect estimate was attenuated and no longer statistically significant (HR = 1.174, 95% CI: 0.901–1.529, *p* = 0.234), although the direction of the association remained consistent with the primary analysis (Supplementary Tables S6 and S7).

## 4. Discussion

### 4.1. Principal Findings

In this retrospective cohort study based on the MIMIC-IV database, we found that a lower PNI at ICU admission was associated with higher 90-day mortality in multivariable-adjusted Cox models. This association remained significant after adjustment for demographics, comorbidities, illness severity, and major therapeutic interventions and showed an approximately linear, monotonic inverse dose–response pattern. The prognostic effect of PNI was also consistent across multiple clinically relevant subgroups. However, in propensity score-matched analysis, this association was attenuated and no longer statistically significant, suggesting that the independent effect of PNI remains uncertain. Nonetheless, these findings suggest that PNI, a simple composite index derived from serum albumin and lymphocyte count, may serve as a useful adjunctive marker for early risk stratification and prognostic assessment, but its clinical utility requires further prospective validation.

### 4.2. Potential Mechanisms Linking PNI to Outcomes

PNI was originally developed and validated by Onodera et al. [13] as a nutritional–immune index in patients undergoing gastrointestinal cancer surgery. However, in the context of acute critical illness—particularly surgical sepsis complicated by AKI—the interpretation of PNI requires substantial caution. It is important to acknowledge that, in the acute setting, PNI is unlikely to represent a pure measure of chronic nutritional status. Both albumin and lymphocyte count are strongly influenced by acute systemic inflammation, capillary leak, fluid resuscitation, and organ dysfunction. Thus, PNI should be viewed as an integrative marker of nutritional–immune reserve and acute illness burden rather than a specific nutrition index.

In our cohort, low-PNI patients had higher lactate, higher bilirubin, and more frequent CRRT/vasopressor use (Supplementary Table S5), indicating greater illness severity at ICU admission. This suggests that low PNI may partly reflect overall severity rather than isolated malnutrition. Although we adjusted for multiple severity indicators and performed propensity score matching, residual confounding cannot be fully excluded.

To the extent that PNI may partially reflect nutritional–immune reserve beyond acute illness severity, albumin is not only a classical marker of nutritional status but also a negative acute-phase protein in critical illness [26,27]. In sepsis, systemic inflammation and increased capillary permeability promote extravasation of albumin into the interstitium, while hepatic synthesis is suppressed and fluid resuscitation leads to hemodilution. The resulting hypoalbuminemia is closely linked to reduced plasma oncotic pressure, interstitial and pulmonary edema, and increased renal interstitial pressure, all of which can aggravate renal ischemia and hypoperfusion and promote AKI onset and progression [4,28]. Recent database studies have confirmed the prognostic value of albumin-based composite indices. Elevated red cell distribution width-to-albumin ratio (RAR) predicts 30-day mortality in sepsis-associated delirium [29], and lactate-to-albumin ratio (LAR) predicts in-hospital mortality in ICU patients [30]. Similarly, and again recognizing the confounding effects of acute illness, lymphopenia is a well-recognized hallmark of sepsis-induced immunosuppression [31,32]. In addition to sepsis-induced immunosuppression [33], major surgery itself can further impair host immune function, as recent evidence demonstrates that surgical stress induces T-cell immunometabolic paralysis through mitochondrial damage caused by myeloid-derived suppressor cells [34]. In patients who already have AKI, impaired immune function may not only delay renal recovery but also increase susceptibility to secondary infections, thereby worsening overall prognosis [3,5].

By integrating albumin and lymphocyte count, PNI may provide a composite snapshot of a patient’s tolerance to the “dual hit” of malnutrition and immune dysfunction in critical illness. In our study, lower PNI values were associated with a higher risk of 90-day death, which is consistent with the notion that patients with impaired nutritional and immune reserves are less able to withstand the combined insults of surgical trauma, sepsis, and AKI. The near-linear inverse relationship observed in the restricted cubic spline analysis suggests a continuous gradient of risk rather than a distinct threshold, implying that even modest improvements in PNI may confer incremental survival benefit.

### 4.3. Comparison with Previous Studies

Previous studies have reported associations between PNI and outcomes in general sepsis or mixed ICU populations, with lower PNI consistently linked to higher short- and long-term mortality [16,17,18,19]. However, evidence specific to patients with both surgical sepsis and AKI has been limited, often derived from single-center, small-sample cohorts without detailed dose–response or subgroup analyses. Our study adds to the literature by focusing on a clearly defined, particularly vulnerable subgroup: patients admitted to surgical or surgery-related ICUs, fulfilling Sepsis-3 criteria and meeting KDIGO definitions for AKI. This group typically experiences the combined effects of perioperative trauma, unstable hemodynamics, infection, and nephrotoxic exposures. Using a large, real-world dataset, we demonstrated that baseline PNI retains prognostic relevance in this complex clinical setting and provides systematic evidence regarding its dose–response relationship and stability across subpopulations. The linear relationship between PNI and mortality in our surgical sepsis–AKI cohort differs from the U-shaped association of TyG-BMI reported in general sepsis [35], underscoring the unique prognostic value of nutritional–immune reserve in this high-risk subgroup.

Methodologically, our work offers several strengths. First, we treated PNI as a continuous variable and used restricted cubic splines to explore the shape of the association with mortality, rather than arbitrarily dichotomizing at the median. The approximately linear pattern we observed argues against a single “magic” cut-off and supports using PNI to capture gradations of risk. Second, we applied the minimum *p*-value method to derive an optimal cut-off (PNI = 29.51) for risk stratification, under constraints on group size, and confirmed clear separation of Kaplan–Meier survival curves between low- and high-PNI groups. Third, the protective association of higher PNI persisted after extensive adjustment for confounders, including age, comorbidities, SOFA score, lactate, creatinine, and major interventions (vasopressors and CRRT). Finally, subgroup analyses showed generally consistent HR estimates across most strata, with the only significant interaction observed for illness severity (SOFA score, *p* for interaction = 0.026). Specifically, the association between low PNI and 90-day mortality was stronger in patients with lower SOFA scores (HR = 2.00) than in those with higher SOFA scores (HR = 1.28). No other significant interactions were detected, supporting the overall generalizability of PNI as a prognostic marker across diverse subgroups. This finding aligns with a broader principle: biomarker meaning depends on patient context. A large ICU registry study showed that leukocytosis predicted mortality only in non-infected patients, disappearing in those with infection—reflecting an adaptive response [36]. Similarly, the protective effect of higher PNI was stronger in patients with lower SOFA scores, supporting context-specific biomarker interpretation in sepsis.

While the cut-off of 29.51 may assist in risk stratification within this cohort, this threshold was derived from the same dataset using the minimum *p*-value method and has not been externally validated. Clinicians should interpret this value with caution and consider it as one of several parameters in the overall clinical assessment.

The modest effect size observed in this study warrants discussion regarding its clinical utility. The per-1-point HR of 0.985 translates to approximately a 1.5% reduction in mortality risk per unit increase in PNI, and the per-5-point HR of 0.927 corresponds to a 7.3% reduction per 5-point increment—an effect that is small at the individual level. However, at the population level, even a modest effect may be meaningful for early risk stratification when applied to a high-risk population such as surgical sepsis with AKI. Nevertheless, the upper bound of the 95% confidence interval approaching unity (0.997) suggests that the precision of this estimate is limited, and the effect should not be overinterpreted as a strong or definitive prognostic signal. We therefore view PNI not as a standalone predictor but as one of several readily available clinical parameters that may collectively inform risk assessment.

However, the incremental predictive value of PNI beyond established risk factors (e.g., SOFA score, lactate, comorbidities) was not formally evaluated in this study and remains to be clarified in future work.

### 4.4. Clinical Implications for Nutritional and Immune Management

From a clinical perspective, PNI is attractive because it is simple to calculate, relies only on routine laboratory tests, and does not require additional costs or specialized assays. In resource-limited settings or at the point of care, PNI could be incorporated into early assessment at ICU admission to identify patients at high risk of adverse outcomes. Based on our findings, several potential applications can be envisioned.

First, PNI may serve as an early risk stratification tool. A low baseline PNI (e.g., below approximately 29.5) identifies patients with compromised nutritional and immune reserves who may be more vulnerable to prolonged organ dysfunction and death. These patients may benefit from closer hemodynamic and renal monitoring, more frequent reassessment, and prompt escalation of supportive therapies.

Second, while PNI may help identify patients with compromised nutritional–immune reserve, whether PNI-guided nutritional or immune-modulating interventions can improve outcomes remains unknown and requires prospective interventional studies. At present, PNI should be viewed as an exploratory risk stratification tool rather than a direct guide for therapy. Nonetheless, the underlying rationale—that nutritional status and immune function are interconnected in critical illness—remains supported by existing evidence [10,11], and ongoing research continues to explore targeted immunomodulatory approaches in sepsis [37,38].

Third, PNI may complement traditional severity scores in comprehensive prognostic assessment. Tools such as SOFA and APACHE scores primarily capture current organ dysfunction, whereas PNI reflects the host’s underlying biological reserves. In surgical sepsis with AKI, where patients often oscillate between phases of hyperinflammation and immunosuppression and are exposed to repeated surgical and hemodynamic stressors, combining PNI with organ failure scores may potentially improve risk prediction, but this requires formal evaluation in future studies.

The contemporary understanding of sepsis has shifted from rigid protocols to phenotype-driven, individualized care [39]. In this framework, early standardized interventions remain essential, but later phases require patient-tailored strategies—particularly for complex subgroups such as surgical sepsis with AKI. Our findings suggest that PNI may provide additional prognostic information beyond acute organ failure, although this incremental value was not formally quantified in our study. By identifying patients with low nutritional–immune reserves, PNI may assist in identifying patients who could benefit from closer monitoring, thus operationalizing a staged, physiology-guided management approach.

### 4.5. Limitations

Several limitations should be acknowledged. First, residual confounding is possible in any retrospective observational study, and unmeasured factors such as pre-existing nutritional status, frailty, or intraoperative details were not available in the MIMIC-IV database. Second, PNI was assessed only once at ICU admission, and dynamic changes during hospitalization were not captured [40,41]. Third, the data originate from a single U.S. tertiary center, which may limit generalizability to other populations or healthcare settings. Fourth, causality cannot be established due to the observational design. Moreover, a substantial number of patients were excluded due to missing albumin or lymphocyte count measurements within the first 24 h after ICU admission. While this exclusion was necessary for PNI calculation, it may have introduced selection bias. We were unable to formally compare included and excluded patients because data for excluded patients were not retained in our extraction. Nonetheless, it is plausible that patients with missing laboratory measurements differed from those with complete data—for example, they may have had more severe illness, more rapid clinical deterioration, or less frequent laboratory monitoring. If this were the case, our findings may not be fully generalizable to the most severely ill patients or to settings where routine laboratory testing is less readily available. Future studies with more complete data collection are needed to confirm our results. In addition, although the association was significant in multivariable models, it was attenuated and no longer statistically significant in the fully adjusted propensity score-matched analysis, suggesting that the independent effect of PNI should be interpreted with caution. Finally, SOFA scores were calculated using the worst values within the first 24 h after ICU admission, whereas AKI was defined using KDIGO criteria over the ICU stay. Therefore, the admission SOFA may not fully reflect the peak severity of AKI or organ dysfunction that developed later during the ICU stay.

## 5. Conclusions

In this large real-world cohort derived from the MIMIC-IV database, lower PNI at ICU admission was consistently associated with higher 90-day all-cause mortality in patients with surgical sepsis and AKI. This association appeared approximately linear and remained significant in multivariable analyses adjusting for a wide range of confounders and major therapeutic interventions and was broadly consistent across key clinical subgroups. However, in the propensity score-matched analysis, the association was attenuated and no longer statistically significant, suggesting that the independent effect of PNI on mortality remains uncertain and should be interpreted with caution. Given its simplicity, low cost, and wide availability from routine laboratory tests, PNI may serve as a simple adjunctive marker for early risk stratification and prognostic evaluation in this high-risk population. Future prospective studies are warranted to validate these findings and to determine whether dynamic PNI monitoring can provide additional prognostic information beyond that captured by acute illness severity.

## Figures and Tables

**Table 1:** Baseline characteristics of patients with surgical sepsis and AKI, stratified by 90-day outcome (selected variables).

Characteristic	Overall (N = 1483) ^1^	90-Day Survivor (N = 1035) ^1^	90-Day Non-Survivor (N = 448) ^1^	*p* Value ^2^
Sex				0.143
Female	587 (40%)	397 (38%)	190 (42%)	
Male	896 (60%)	638 (62%)	258 (58%)	
Age (years)	65 (54, 76)	64 (53, 74)	70 (57, 82)	<0.001
Weight	82 (70, 97)	83 (71, 98)	78 (65, 94)	<0.001
Race				0.006
White	882 (59%)	635 (61%)	247 (55%)	
Black	144 (9.7%)	103 (10.0%)	41 (9.2%)	
Asian	48 (3.2%)	38 (3.7%)	10 (2.2%)	
Other	409 (28%)	259 (25%)	150 (33%)	
Heart rate	91 (79, 106)	91 (78, 105)	92 (81, 111)	0.012
Respiratory rate	19 (16, 24)	18 (16, 23)	20 (16, 24)	<0.001
Albumin	3.00 (2.50, 3.50)	3.00 (2.50, 3.50)	2.90 (2.40, 3.50)	0.016
Lactate	1.80 (1.20, 3.10)	1.70 (1.10, 2.60)	2.50 (1.50, 5.10)	<0.001
Absolute lymphocyte count	1.02 (0.63, 1.63)	1.07 (0.65, 1.71)	0.90 (0.58, 1.42)	<0.001
SOFA score	2.00 (0.00, 5.00)	2.00 (0.00, 5.00)	2.00 (1.00, 5.00)	0.002
CRRT				<0.001
No	1257 (85%)	933 (90%)	324 (72%)	
Yes	226 (15%)	102 (9.9%)	124 (28%)	
Vasoactive drugs				<0.001
No	589 (40%)	447 (43%)	142 (32%)	
Yes	894 (60%)	588 (57%)	306 (68%)	
PNI	36 (30, 42)	36 (30, 43)	35 (29, 41)	<0.001

^1^ Data are presented as n (%) or median (Q1–Q3), as appropriate. ^2^ *p* values were calculated using Pearson’s chi-squared test or the Wilcoxon rank-sum test, as appropriate. CRRT, continuous renal replacement therapy; PNI, prognostic nutritional index; SOFA, Sequential Organ Failure Assessment.

**Table 2:** Multivariable Cox regression analysis of the association between PNI and 90-day mortality.

Endpoint	Model	Exposure	HR	95% CI	*p* Value
90-day mortality	Model 1	PNI per 1-point increase	0.981	0.970–0.992	<0.001
90-day mortality	Model 2	PNI per 1-point increase	0.978	0.967–0.989	<0.001
90-day mortality	Model 3	PNI per 1-point increase	0.985	0.973–0.996	0.008
90-day mortality	Model 4	PNI per 1-point increase	0.985	0.974–0.997	0.012

The table shows hazard ratios (HRs) with 95% confidence intervals (CIs) for PNI (per 1-point increase) across four sequential models. PNI remained significantly associated with lower 90-day mortality in all models. CI, confidence interval; HR, hazard ratio; PNI, prognostic nutritional index.

**Table 3:** Quartile analysis of the PNI for 90-day all-cause mortality.

Endpoint	Analysis	Category	Reference	HR	95% CI	*p* Value
90-day mortality	Survival-derived cutoff	Low PNI vs. High PNI	High PNI	1.351	1.096–1.666	0.005
90-day mortality	PNI quartiles	Q3	Q4 highest	1.137	0.856–1.508	0.375
90-day mortality	PNI quartiles	Q2	Q4 highest	1.129	0.849–1.500	0.404
90-day mortality	PNI quartiles	Q1 lowest	Q4 highest	1.424	1.079–1.879	0.013
90-day mortality	PNI quartile trend	Per one higher PNI quartile	NA	0.897	0.821–0.980	0.016

Data are presented as hazard ratios (HRs) with 95% confidence intervals (CIs) from multivariable Cox regression models (adjusted for age, sex, race, SOFA score, lactate, comorbidities, vasopressors, and CRRT). The survival-derived cut-off was 29.51 (low PNI < 29.51 vs. high PNI ≥ 29.51). Quartiles were defined based on the PNI distribution (Q1 lowest to Q4 highest). PNI quartile trend represents the HR per one-quartile increase. NA indicates not applicable. CI, confidence interval; HR, hazard ratio; NA, not applicable; PNI, prognostic nutritional index.

## Data Availability

The data were extracted from the Medical Information Mart for Intensive Care IV (MIMIC-IV, version 3.1) database. All patient data in MIMIC-IV are de-identified, and the privacy of patients was fully protected. Therefore, the need for additional informed consent was waived by the institutional ethics committee of Beth Israel Deaconess Medical Center.

## Supplementary Materials

The following supporting information can be downloaded at: https://www.mdpi.com/article/10.3390/jcm15145706/s1, Table S1. Complete baseline characteristics of patients with surgical sepsis and AKI, stratified by 90-day outcome; Table S2. Univariable Cox regression analysis of factors associated with 90-day mortality; Table S3. Test of proportional hazards assumption for the multivariable Cox models (Model 3 and Model 4); Table S4. Variance inflation factor (VIF) for covariates in the multivariable Cox models (Model 3 and Model 4); Table S5. Baseline characteristics of patients stratified by the PNI cut-off of 29.51 (low-PNI vs. high-PNI groups); Table S6. Standardized mean differences (SMDs) of covariates before and after 1:1 propensity score matching; Table S7. Cox regression results for low PNI and 90-day mortality before and after propensity score matching; Figure S1. Kaplan–Meier survival curves for 90-day mortality according to PNI quartiles; Figure S2. Love plot of standardized mean differences (SMDs) before and after propensity score matching.

## Abbreviations

The following abbreviations are used in this manuscript:

AKI	Acute kidney injury
CI	Confidence interval
CRRT	Continuous renal replacement therapy
HR	Hazard ratio
ICU	Intensive care unit
MIMIC-IV	Medical Information Mart for Intensive Care IV
PNI	Prognostic nutritional index
PSM	Propensity score matching
RCS	Restricted cubic splines
SMD	Standardized mean difference
SOFA	Sequential Organ Failure Assessment
STROBE	Strengthening the Reporting of Observational Studies in Epidemiology
VIF	Variance inflation factor
