# Supplementary material for: Prognostic Nutritional Index as a Predictor of 90-Day Mortality in Surgical Sepsis Patients with Acute Kidney Injury—A Retrospective Cohort Study Based on the MIMIC-IV Database
Source: J Clin Med. 2026 Jul 21;15(14):5706. doi: 10.3390/jcm15145706 (PMC13413065; doi:10.3390/jcm15145706)
Supplement: Supplementary file 1 [file jcm-15-05706-s001.zip › jcm-4425332-supplementary.pdf]

Supplementary Table S1. Complete baseline characteristics of patients with surgical sepsis and AKI, stratified by 90-day outcome.

Characteristic	Overall (*n* = 1483)[1]	90-day survivor (*n* = 1035)[1]	90-day non-survivor (*n* = 448)[1]	*p* value[2]
Sex				0.143
Female	587 (40%)	397 (38%)	190 (42%)	
Male	896 (60%)	638 (62%)	258 (58%)	
Age (years)	65 (54, 76)	64 (53, 74)	70 (57, 82)	<0.001
Weight	82 (70, 97)	83 (71, 98)	78 (65, 94)	<0.001
Race				0.006
White	882 (59%)	635 (61%)	247 (55%)	
Black	144 (9.7%)	103 (10.0%)	41 (9.2%)	
Asian	48 (3.2%)	38 (3.7%)	10 (2.2%)	
Other	409 (28%)	259 (25%)	150 (33%)	
Heart rate	91 (79, 106)	91 (78, 105)	92 (81, 111)	0.012
SpO_2	98.0 (95.0, 100.0)	98.0 (95.0, 100.0)	97.0 (94.0, 100.0)	0.001
Mean blood pressure	83 (71, 95)	83 (71, 95)	82 (69, 94)	0.234
Respiratory rate	19 (16, 24)	18 (16, 23)	20 (16, 24)	<0.001
Temperature	36.78 (36.39, 37.17)	36.83 (36.44, 37.22)	36.67 (36.33, 37.00)	<0.001
Total bilirubin	0.80 (0.40, 1.70)	0.80 (0.40, 1.70)	0.80 (0.50, 1.90)	0.107
AST	51 (27, 145)	51 (27, 135)	52 (28, 155)	0.316

Characteristic	Overall (n = 1483)[1]	90-day survivor (n = 1035)[1]	90-day non-survivor (n = 448)[1]	p value[2]
ALT	31 (18, 81)	31 (18, 86)	32 (18, 73)	0.895
Albumin	3.00 (2.50, 3.50)	3.00 (2.50, 3.50)	2.90 (2.40, 3.50)	0.016
Lactate	1.80 (1.20, 3.10)	1.70 (1.10, 2.60)	2.50 (1.50, 5.10)	<0.001
BUN	21 (15, 35)	20 (14, 31)	27 (17, 44)	<0.001
Creatinine	1.10 (0.80, 1.80)	1.00 (0.80, 1.50)	1.30 (0.90, 2.30)	<0.001
WBC	11 (8, 17)	11 (7, 17)	13 (9, 18)	<0.001
Absolute lymphocyte count	1.02 (0.63, 1.63)	1.07 (0.65, 1.71)	0.90 (0.58, 1.42)	<0.001
RBC	3.62 (2.99, 4.25)	3.65 (2.99, 4.28)	3.57 (2.96, 4.19)	0.235
Hemoglobin	10.80 (9.00, 12.70)	10.80 (9.00, 12.80)	10.70 (8.90, 12.40)	0.268
Platelet	186 (126, 264)	192 (127, 269)	179 (121, 257)	0.108
RDW	14.60 (13.60, 16.40)	14.40 (13.50, 16.00)	15.00 (13.80, 17.10)	<0.001
PT	15 (13, 19)	15 (13, 18)	16 (13, 21)	<0.001
PTT	33 (28, 44)	33 (28, 43)	33 (28, 47)	0.266
INR	1.30 (1.20, 1.70)	1.30 (1.20, 1.70)	1.40 (1.20, 1.90)	<0.001
Bicarbonate	22.0 (19.0, 25.0)	22.0 (19.0, 25.0)	21.0 (17.0, 24.0)	<0.001
Calcium	8.30 (7.80, 8.90)	8.30 (7.80, 8.90)	8.30 (7.75, 8.90)	0.599
Potassium	4.20 (3.80, 4.70)	4.20 (3.80, 4.60)	4.30 (3.80, 4.80)	0.017
Sodium	138.0 (135.0, 141.0)	138.0 (136.0, 141.0)	138.5 (135.0, 142.0)	0.969

Characteristic	Overall (*n* = 1483)[1]	90-day survivor (*n* = 1035)[1]	90-day non-survivor (*n* = 448)[1]	*p* value[2]
Chloride	103 (99, 107)	104 (100, 107)	102 (98, 107)	0.005
Glucose	138 (108, 186)	138 (109, 184)	138 (105, 188)	0.879
SOFA score	2.00 (0.00, 5.00)	2.00 (0.00, 5.00)	2.00 (1.00, 5.00)	0.002
Mechanical ventilation				0.510
No	50 (3.4%)	37 (3.6%)	13 (2.9%)	
Yes	1433 (97%)	998 (96%)	435 (97%)	
CRRT				<0.001
No	1257 (85%)	933 (90%)	324 (72%)	
Yes	226 (15%)	102 (9.9%)	124 (28%)	
Vasoactive drugs				<0.001
No	589 (40%)	447 (43%)	142 (32%)	
Yes	894 (60%)	588 (57%)	306 (68%)	
Myocardial infarction				0.591
No	1204 (81%)	844 (82%)	360 (80%)	
Yes	279 (19%)	191 (18%)	88 (20%)	
Congestive heart failure				0.038
No	1096 (74%)	781 (75%)	315 (70%)	
Yes	387 (26%)	254 (25%)	133 (30%)	

**Characteristic**	**Overall (n = 1483)**[1]	**90-day survivor (n = 1035)**[1]	**90-day non-survivor (n = 448)**[1]	***p* value**[2]
Cerebrovascular disease				<0.001
No	1232 (83%)	901 (87%)	331 (74%)	
Yes	251 (17%)	134 (13%)	117 (26%)	
Chronic pulmonary disease				0.068
No	1166 (79%)	827 (80%)	339 (76%)	
Yes	317 (21%)	208 (20%)	109 (24%)	
Diabetes				0.488
No	1034 (70%)	716 (69%)	318 (71%)	
Yes	449 (30%)	319 (31%)	130 (29%)	
Malignant cancer				0.156
No	1311 (88%)	923 (89%)	388 (87%)	
Yes	172 (12%)	112 (11%)	60 (13%)	
Severe liver disease				0.450
No	1262 (85%)	876 (85%)	386 (86%)	
Yes	221 (15%)	159 (15%)	62 (14%)	
PNI	36 (30, 42)	36 (30, 43)	35 (29, 41)	<0.001

[1]Data are presented as *n* (%) or median (Q1-Q3), as appropriate.

Characteristic	Overall (n = 1483)[1]	90-day survivor (n = 1035)[1]	90-day non-survivor (n = 448)[1]	p value[2]

[2] p values were calculated using Pearson's chi-squared test or the Wilcoxon rank-sum test, as appropriate.

AKI, acute kidney injury; ALT, alanine aminotransferase; AST, aspartate aminotransferase; BUN, blood urea nitrogen; CRRT, continuous renal replacement therapy; INR, international normalized ratio; PNI, prognostic nutritional index; PT, prothrombin time; PTT, partial thromboplastin time; RBC, red blood cell count; RDW, red cell distribution width; SOFA, Sequential Organ Failure Assessment; SpO_2, oxygen saturation; WBC, white blood cell count.

Supplementary Table S2. Univariable Cox regression analysis of factors associated with 90-day mortality.

Endpoint	Variable	HR	95% CI	p value
90-day mortality	Sex	0.868	0.719-1.046	0.138
90-day mortality	Age (years)	1.020	1.014-1.027	<0.001
90-day mortality	Weight	0.992	0.988-0.996	<0.001
90-day mortality	Race (Black)	1.040	0.748-1.448	0.815
90-day mortality	Race (Asian)	0.703	0.373-1.322	0.274
90-day mortality	Race (Other)	1.440	1.175-1.764	<0.001
90-day mortality	Heart rate	1.005	1.001-1.009	<0.05
90-day mortality	SpO_2	0.960	0.944-0.976	<0.001

Endpoint	Variable	HR	95% CI	*p* value
90-day mortality	Mean blood pressure	0.997	0.992-1.002	0.211
90-day mortality	Respiratory rate	1.022	1.009-1.036	<0.05
90-day mortality	Temperature	0.805	0.748-0.866	<0.001
90-day mortality	Total bilirubin	1.018	1.005-1.032	<0.05
90-day mortality	AST	1.000	1.000-1.000	0.077
90-day mortality	ALT	1.000	1.000-1.000	0.103
90-day mortality	Albumin	0.857	0.750-0.980	<0.05
90-day mortality	Lactate	1.178	1.151-1.206	<0.001
90-day mortality	BUN	1.008	1.005-1.011	<0.001
90-day mortality	Creatinine	1.091	1.040-1.145	<0.001
90-day mortality	WBC	1.021	1.010-1.032	<0.001
90-day mortality	Absolute lymphocyte count	0.819	0.725-0.924	<0.05
90-day mortality	RBC	0.961	0.865-1.068	0.461
90-day mortality	Hemoglobin	0.987	0.952-1.024	0.496
90-day mortality	Platelet	0.999	0.999-1.000	0.140
90-day mortality	RDW	1.069	1.035-1.103	<0.001
90-day mortality	PT	1.015	1.009-1.022	<0.001

Endpoint	Variable	HR	95% CI	*p* value
90-day mortality	PTT	1.002	0.999-1.005	0.194
90-day mortality	INR	1.180	1.091-1.276	<0.001
90-day mortality	Bicarbonate	0.949	0.931-0.967	<0.001
90-day mortality	Calcium	0.942	0.861-1.030	0.187
90-day mortality	Potassium	1.156	1.040-1.285	<0.05
90-day mortality	Sodium	1.006	0.988-1.023	0.526
90-day mortality	Chloride	0.982	0.969-0.996	<0.05
90-day mortality	Glucose	1.001	1.000-1.002	<0.05
90-day mortality	SOFA score	1.054	1.024-1.084	<0.001
90-day mortality	Mechanical ventilation (Yes)	1.215	0.700-2.109	0.489
90-day mortality	CRRT (Yes)	2.645	2.149-3.254	<0.001
90-day mortality	Vasoactive drugs (Yes)	1.551	1.271-1.893	<0.001
90-day mortality	Myocardial infarction (Yes)	1.046	0.829-1.321	0.705
90-day mortality	Congestive heart failure (Yes)	1.212	0.990-1.485	0.063
90-day mortality	Cerebrovascular disease (Yes)	2.009	1.627-2.481	<0.001
90-day mortality	Chronic pulmonary disease (Yes)	1.219	0.982-1.513	0.072

Endpoint	Variable	HR	95% CI	*p* value
90-day mortality	Diabetes (Yes)	0.911	0.743-1.117	0.369
90-day mortality	Malignant cancer (Yes)	1.155	0.880-1.516	0.299
90-day mortality	Severe liver disease (Yes)	0.884	0.676-1.156	0.367
90-day mortality	PNI	0.981	0.970-0.992	<0.001

Hazard ratios (HRs) and 95% confidence intervals (CIs) were calculated using univariable Cox proportional hazards regression. ALT, alanine aminotransferase; AST, aspartate aminotransferase; BUN, blood urea nitrogen; CRRT, continuous renal replacement therapy; INR, international normalized ratio; PNI, prognostic nutritional index; PT, prothrombin time; PTT, partial thromboplastin time; RBC, red blood cell count; RDW, red cell distribution width; SOFA, Sequential Organ Failure Assessment; SpO_2, oxygen saturation; WBC, white blood cell count.

Supplementary Table S3. Test of proportional hazards assumption for the multivariable Cox models (Model 3 and Model 4).

Endpoint	Model	Variable	χ^2	df	*p* value
90-day mortality	Model 4	PNI	2.872578172	1	0.090
90-day mortality	Model 4	Age	12.387560241	1	0.0004
90-day mortality	Model 4	Gender	0.408031719	1	0.523
90-day mortality	Model 4	Race	6.757045654	3	0.080
90-day mortality	Model 4	WBC	0.475779531	1	0.490

Endpoint	Model	Variable	χ^2	df	*p* value
90-day mortality	Model 4	Lactate	0.222665549	1	0.637
90-day mortality	Model 4	SOFA	1.611061074	1	0.204
90-day mortality	Model 4	Congestive heart failure	7.088310031	1	0.008
90-day mortality	Model 4	Diabetes	5.139227074	1	0.023
90-day mortality	Model 4	Cerebrovascular disease	0.004300305	1	0.948
90-day mortality	Model 4	Chronic pulmonary disease	0.251219207	1	0.616
90-day mortality	Model 4	Vasoactive	3.530629213	1	0.060
90-day mortality	Model 4	CRRT	2.752340941	1	0.097
90-day mortality	Model 4	GLOBAL	41.064394864	15	0.0003
90-day mortality	Model 3	PNI	3.031036129	1	0.082
90-day mortality	Model 3	Age	13.404767221	1	0.0003
90-day mortality	Model 3	Gender	0.269760126	1	0.603
90-day mortality	Model 3	Race	7.799204351	3	0.050
90-day mortality	Model 3	WBC	0.772447397	1	0.379
90-day mortality	Model 3	Lactate	1.378243776	1	0.240
90-day mortality	Model 3	SOFA	1.405950705	1	0.236
90-day mortality	Model 3	Congestive heart failure	6.433614991	1	0.011

Endpoint	Model	Variable	χ^2	df	*p* value
90-day mortality	Model 3	Diabetes	4.875323169	1	0.027
90-day mortality	Model 3	Cerebrovascular disease	0.017969155	1	0.893
90-day mortality	Model 3	Chronic pulmonary disease	0.150142081	1	0.698
90-day mortality	Model 3	GLOBAL	33.933506723	13	0.001

The proportional hazards assumption was tested using Schoenfeld residuals, with both variable-specific and global tests reported.

Model 3 was adjusted for age, sex, race, SOFA score, lactate, white blood cell count (WBC), and major comorbidities (congestive heart failure, diabetes, cerebrovascular disease, chronic pulmonary disease). Model 4 was further adjusted for use of vasoactive drugs and continuous renal replacement therapy (CRRT). CRRT, continuous renal replacement therapy; df, degrees of freedom; PNI, prognostic nutritional index; SOFA, Sequential Organ Failure Assessment; WBC, white blood cell count.

Supplementary Table S4. Variance inflation factor (VIF) for covariates in the multivariable Cox models (Model 3 and Model 4).

Model	Variable	GVIF	df	Adjusted GVIF
Model 4	PNI	1.094272	1	1.046075
Model 4	Age	1.111232	1	1.054150
Model 4	Gender	1.042513	1	1.021035
Model 4	Race	1.063401	3	1.010298

Model	Variable	GVIF	df	Adjusted GVIF
Model 4	WBC	1.055515	1	1.027383
Model 4	Lactate	1.172168	1	1.082667
Model 4	SOFA	1.186389	1	1.089215
Model 4	Congestive heart failure	1.098210	1	1.047955
Model 4	Diabetes	1.058052	1	1.028617
Model 4	Cerebrovascular disease	1.049344	1	1.024375
Model 4	Chronic pulmonary disease	1.057867	1	1.028527
Model 4	Vasoactive	1.077916	1	1.038227
Model 4	CRRT	1.176478	1	1.084656
Model 3	PNI	1.092398	1	1.045179
Model 3	Age	1.107181	1	1.052227
Model 3	Gender	1.042448	1	1.021003
Model 3	Race	1.047535	3	1.007770
Model 3	WBC	1.048443	1	1.023935
Model 3	Lactate	1.127336	1	1.061761
Model 3	SOFA	1.110730	1	1.053912
Model 3	Congestive heart failure	1.091934	1	1.044956

Model	Variable	GVIF	df	Adjusted GVIF
Model 3	Diabetes	1.056949	1	1.028080
Model 3	Cerebrovascular disease	1.049154	1	1.024282
Model 3	Chronic pulmonary disease	1.051698	1	1.025523

Generalized variance inflation factor (GVIF) and adjusted GVIF are shown. An adjusted GVIF < 5 is commonly considered to indicate no significant multicollinearity. CRRT, continuous renal replacement therapy; df, degrees of freedom; PNI, prognostic nutritional index; SOFA, Sequential Organ Failure Assessment; WBC, white blood cell count.

Supplementary Table S5. Baseline characteristics of patients stratified by the PNI cut-off of 29.51 (low-PNI vs. high-PNI groups).

Characteristic	Overall (*n* = 1483)[1]	Low PNI (*n* = 354)[1]	High PNI (*n* = 1129)[1]	*p* value[2]
Sex				0.006
Female	587 (40%)	162 (46%)	425 (38%)	
Male	896 (60%)	192 (54%)	704 (62%)	
Age (years)	65 (54, 76)	64 (53, 76)	65 (54, 76)	0.535
Weight	82 (70, 97)	79 (67, 94)	82 (70, 98)	0.004
Race				0.721
White	882 (59%)	218 (62%)	664 (59%)	
Black	144 (9.7%)	34 (9.6%)	110 (9.7%)	

Characteristic	Overall (*n* = 1483)[1]	Low PNI (*n* = 354)[1]	High PNI (*n* = 1129)[1]	*p* value[2]
Asian	48 (3.2%)	9 (2.5%)	39 (3.5%)	
Other	409 (28%)	93 (26%)	316 (28%)	
Heart rate	91 (79, 106)	97 (84, 112)	90 (77, 104)	<0.001
SpO_2	98.0 (95.0, 100.0)	98.0 (95.0, 100.0)	98.0 (95.0, 100.0)	0.294
Mean blood pressure	83 (71, 95)	81 (69, 91)	83 (72, 96)	<0.001
Respiratory rate	19 (16, 24)	20 (16, 24)	19 (16, 23)	0.074
Temperature	36.78 (36.39, 37.17)	36.78 (36.39, 37.17)	36.78 (36.44, 37.17)	0.441
Total bilirubin	0.80 (0.40, 1.70)	1.00 (0.50, 2.50)	0.80 (0.40, 1.60)	<0.001
AST	51 (27, 145)	62 (32, 178)	48 (27, 133)	0.002
ALT	31 (18, 81)	38 (19, 127)	30 (17, 74)	0.005
Albumin	3.00 (2.50, 3.50)	2.30 (2.00, 2.50)	3.20 (2.90, 3.70)	<0.001
Lactate	1.80 (1.20, 3.10)	2.30 (1.40, 3.90)	1.70 (1.20, 2.80)	<0.001
BUN	21 (15, 35)	24 (14, 42)	21 (15, 33)	0.009
Creatinine	1.10 (0.80, 1.80)	1.20 (0.80, 2.00)	1.10 (0.80, 1.70)	0.408
WBC	11 (8, 17)	11 (6, 17)	11 (8, 17)	0.150
Absolute lymphocyte count	1.02 (0.63, 1.63)	0.68 (0.42, 0.97)	1.19 (0.74, 1.82)	<0.001
RBC	3.62 (2.99, 4.25)	3.26 (2.72, 3.80)	3.74 (3.11, 4.35)	<0.001
Hemoglobin	10.80 (9.00, 12.70)	9.90 (8.30, 11.30)	11.20 (9.30, 13.00)	<0.001

Characteristic	Overall (n = 1483)[1]	Low PNI (n = 354)[1]	High PNI (n = 1129)[1]	p value[2]
Platelet	186 (126, 264)	170 (101, 273)	189 (134, 261)	0.028
RDW	14.60 (13.60, 16.40)	15.35 (14.10, 17.30)	14.40 (13.40, 16.00)	<0.001
PT	15 (13, 19)	17 (14, 22)	14 (13, 18)	<0.001
PTT	33 (28, 44)	36 (30, 47)	32 (28, 42)	<0.001
INR	1.30 (1.20, 1.70)	1.50 (1.30, 2.00)	1.30 (1.10, 1.60)	<0.001
Bicarbonate	22.0 (19.0, 25.0)	20.0 (17.0, 23.0)	22.0 (19.0, 25.0)	<0.001
Calcium	8.30 (7.80, 8.90)	7.90 (7.30, 8.50)	8.40 (7.90, 9.00)	<0.001
Potassium	4.20 (3.80, 4.70)	4.10 (3.70, 4.60)	4.20 (3.80, 4.70)	0.409
Sodium	138.0 (135.0, 141.0)	138.0 (135.0, 142.0)	139.0 (135.0, 141.0)	0.750
Chloride	103 (99, 107)	105 (100, 109)	103 (99, 107)	<0.001
Glucose	138 (108, 186)	138 (104, 184)	138 (109, 186)	0.353
SOFA score	2.00 (0.00, 5.00)	3.00 (1.00, 6.00)	2.00 (0.00, 4.00)	<0.001
Mechanical ventilation				0.752
No	50 (3.4%)	11 (3.1%)	39 (3.5%)	
Yes	1433 (97%)	343 (97%)	1090 (97%)	
CRRT				0.007
No	1257 (85%)	284 (80%)	973 (86%)	
Yes	226 (15%)	70 (20%)	156 (14%)	

Characteristic	Overall (n = 1483)[1]	Low PNI (n = 354)[1]	High PNI (n = 1129)[1]	p value[2]
Vasoactive drugs				<0.001
No	589 (40%)	103 (29%)	486 (43%)	
Yes	894 (60%)	251 (71%)	643 (57%)	
Myocardial infarction				<0.001
No	1204 (81%)	309 (87%)	895 (79%)	
Yes	279 (19%)	45 (13%)	234 (21%)	
Congestive heart failure				0.007
No	1096 (74%)	281 (79%)	815 (72%)	
Yes	387 (26%)	73 (21%)	314 (28%)	
Cerebrovascular disease				<0.001
No	1232 (83%)	319 (90%)	913 (81%)	
Yes	251 (17%)	35 (9.9%)	216 (19%)	
Chronic pulmonary disease				0.692
No	1166 (79%)	281 (79%)	885 (78%)	
Yes	317 (21%)	73 (21%)	244 (22%)	
Diabetes				0.003
No	1034 (70%)	269 (76%)	765 (68%)	
Yes	449 (30%)	85 (24%)	364 (32%)	

Characteristic	Overall (n = 1483)[1]	Low PNI (n = 354)[1]	High PNI (n = 1129)[1]	p value[2]
Malignant cancer				0.004
No	1311 (88%)	298 (84%)	1013 (90%)	
Yes	172 (12%)	56 (16%)	116 (10%)	
Severe liver disease				<0.001
No	1262 (85%)	282 (80%)	980 (87%)	
Yes	221 (15%)	72 (20%)	149 (13%)	
PNI	36 (30, 42)	26 (24, 28)	39 (34, 44)	<0.001

[1]Data are presented as n (%) or median (Q1–Q3), as appropriate.

[2]p values were calculated using Pearson's chi-squared test or the Wilcoxon rank-sum test, as appropriate.

ALT, alanine aminotransferase; AST, aspartate aminotransferase; BUN, blood urea nitrogen; CRRT, continuous renal replacement therapy; INR, international normalized ratio; PNI, prognostic nutritional index; PT, prothrombin time; PTT, partial thromboplastin time; RBC, red blood cell count; RDW, red cell distribution width; SOFA, Sequential Organ Failure Assessment; SpO_2, oxygen saturation; WBC, white blood cell count.

Supplementary Table S6. Standardized mean differences (SMDs) of covariates before and after 1:1 propensity score matching.

Variable	Type	Unadjusted SMD	Adjusted SMD	Balanced*

Variable	Type	Unadjusted SMD	Adjusted SMD	Balanced*
distance	Distance	0.822114448	0.022769511	Balanced, <0.1
Age	Contin.	-0.030850437	-0.017539751	Balanced, <0.1
Gender_Male	Binary	-0.081187792	0.005714286	Balanced, <0.1
Race_White	Binary	0.027688120	-0.017142857	Balanced, <0.1
Race_Black	Binary	-0.001386157	0.008571429	Balanced, <0.1
Race_Asian	Binary	-0.009120115	0.002857143	Balanced, <0.1
Race_Other	Binary	-0.017181847	0.005714286	Balanced, <0.1
WBC	Contin.	-0.025706654	-0.005896141	Balanced, <0.1
Lactate	Contin.	0.260780992	0.112127239	Not Balanced, >0.1
SOFA	Contin.	0.365124668	0.041296640	Balanced, <0.1
Congestive heart failure_Yes	Binary	-0.071907543	0.011428571	Balanced, <0.1
Diabetes_Yes	Binary	-0.082296217	0.022857143	Balanced, <0.1
Cerebrovascular disease_Yes	Binary	-0.092449695	0.020000000	Balanced, <0.1
Chronic pulmonary disease_Yes	Binary	-0.009905771	0.005714286	Balanced, <0.1
Vasoactive_Yes	Binary	0.139508990	0.040000000	Balanced, <0.1
CRRT_Yes	Binary	0.059564737	0.031428571	Balanced, <0.1
Ventilator_Yes	Binary	0.003470398	0.014285714	Balanced, <0.1

Variable	Type	Unadjusted SMD	Adjusted SMD	Balanced*
Myocardial infarct_Yes	Binary	-0.080144421	-0.002857143	Balanced, <0.1
Malignant cancer_Yes	Binary	0.055446298	-0.005714286	Balanced, <0.1
Severe liver disease_Yes	Binary	0.071414631	-0.002857143	Balanced, <0.1
Heart rate	Contin.	0.326498040	-0.032384576	Balanced, <0.1
SpO_2	Contin.	-0.097657960	-0.071258970	Balanced, <0.1
MBP	Contin.	-0.179325898	-0.023526075	Balanced, <0.1
Resp_rate	Contin.	0.091979487	-0.019279337	Balanced, <0.1
Temperature	Contin.	-0.100908621	-0.024494007	Balanced, <0.1
Creatinine	Contin.	0.090092271	0.072248289	Balanced, <0.1
Bun	Contin.	0.167250612	0.046970808	Balanced, <0.1
RDW	Contin.	0.387581848	0.055215512	Balanced, <0.1
Hemoglobin	Contin.	-0.554539168	0.003184247	Balanced, <0.1
Platelet	Contin.	-0.031453368	-0.063586145	Balanced, <0.1
Weight	Contin.	-0.148295932	0.040089238	Balanced, <0.1

SMD, standardized mean difference. Unadjusted SMD = before matching; Adjusted SMD = after 1:1 propensity score matching.

* Balanced if SMD < 0.1; otherwise, not balanced. CRRT, continuous renal replacement therapy; MBP, mean blood pressure; RDW, red cell distribution width; SOFA, Sequential Organ Failure Assessment; SpO_2, oxygen saturation; WBC, white blood cell count.

Supplementary Table S7. Cox regression results for low PNI and 90-day mortality before and after propensity score matching.

**Endpoint**	**Cohort**	**Model**	**Comparison**	**HR**	**95% CI**	***p* value**
90-day mortality	Before PSM: full cohort	Model 1	Low PNI vs High PNI	1.509	1.234-1.845	<0.001
90-day mortality	Before PSM: full cohort	Model 2	Low PNI vs High PNI	1.555	1.271-1.903	<0.001
90-day mortality	Before PSM: full cohort	Model 3	Low PNI vs High PNI	1.368	1.107-1.690	0.004
90-day mortality	Before PSM: full cohort	Model 4	Low PNI vs High PNI	1.351	1.096-1.666	0.005
90-day mortality	After PSM: matched cohort	Matched crude Cox	Low PNI vs High PNI	1.314	1.021-1.692	0.034
90-day mortality	After PSM: matched cohort	Matched residual-adjusted Cox	Low PNI vs High PNI	1.174	0.901-1.529	0.234

Models 1-4 correspond to the same sequential adjustments as in the main analysis (Model 1: unadjusted; Model 2: adjusted for age, sex, race; Model 3: further adjusted for SOFA, lactate, WBC, and major comorbidities; Model 4: additionally adjusted for use of vasoactive drugs and CRRT). “Matched crude Cox” denotes an unadjusted Cox analysis in the propensity score-matched cohort. “Matched residual-adjusted Cox” denotes a Cox model further adjusted for lactate and any covariates with a post-matching SMD ≥ 0.1. CI, confidence interval; CRRT, continuous renal replacement therapy; HR, hazard ratio; PNI, prognostic nutritional index; PSM, propensity score matching; SMD, standardized mean difference; SOFA, Sequential Organ Failure Assessment; WBC, white blood cell count.

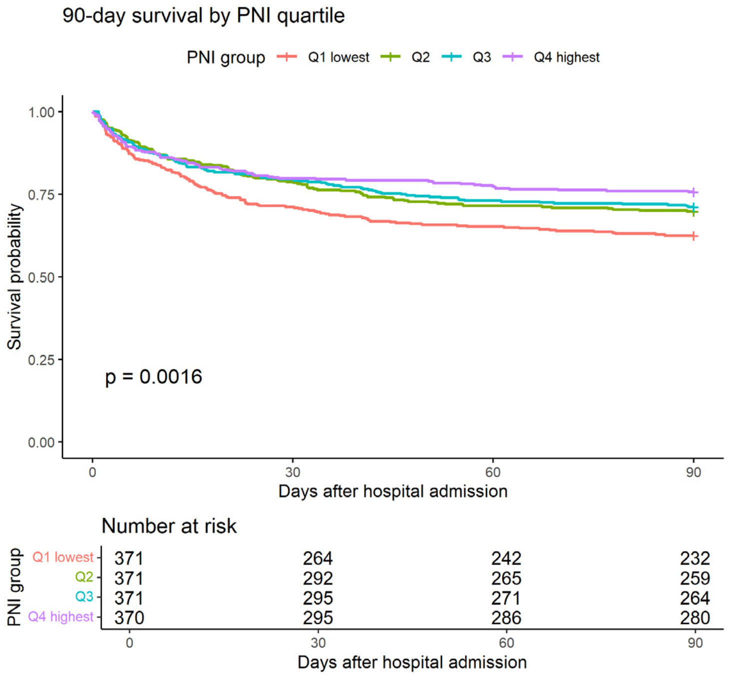

Supplementary Figure S1 Kaplan–Meier survival curves for 90-day mortality according to PNI quartiles.

Patients were divided into four groups based on quartiles of the prognostic nutritional index (PNI) distribution (Q1: lowest; Q4: highest). The survival probability is plotted against days after hospital admission. The numbers of patients at risk at each time interval are displayed below the x-axis. The survival curves demonstrate a graded pattern across quartiles, with the lowest survival in Q1 and the highest in Q4. The log-rank test for trend was significant (*p* for trend = 0.0016), supporting a dose-dependent inverse relationship between PNI and 90-day mortality.

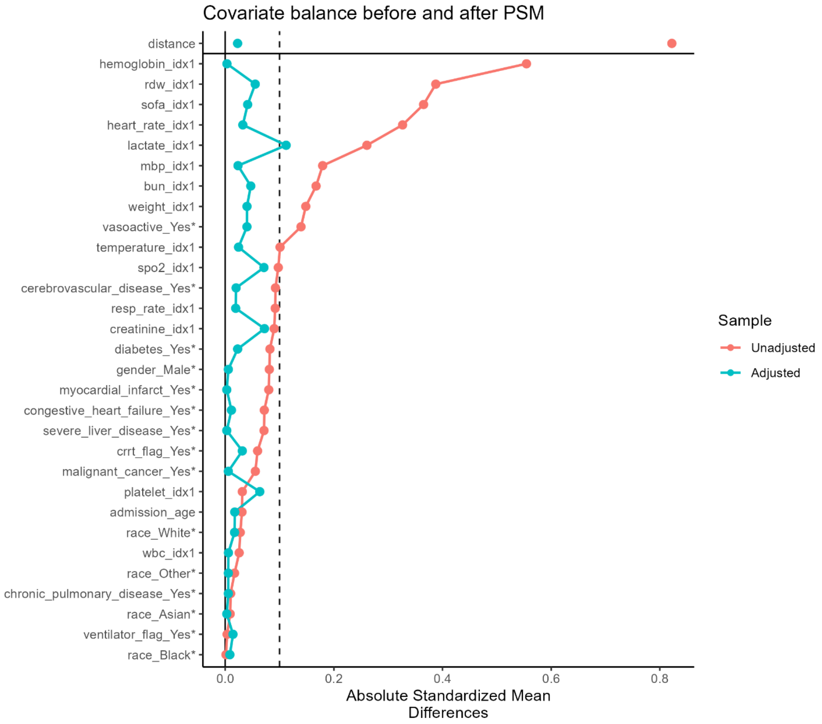

Supplementary Figure S2. Love plot of standardized mean differences (SMDs) before and after propensity score matching.

The plot shows the absolute standardized mean difference (SMD) for each covariate between the low-PNI (PNI < 29.51) and high-PNI (PNI ≥ 29.51) groups before (unadjusted) and after (adjusted) 1:1 propensity score matching. The vertical dashed line indicates the threshold for acceptable balance (SMD < 0.1). After matching, all covariates except lactate (SMD = 0.112) achieved good balance, with SMD values below the 0.1 threshold, confirming that the matching procedure effectively reduced baseline imbalances between the two groups.
